# Machine Learning for Predicting Stroke Risk Stratification Using Multiomics Data: Systematic Review

**DOI:** 10.2196/85654

**Published:** 2026-02-19

**Authors:** Hae Young Yoo, Hyerim Shin, Eun-Jung Kim, Youn-Jung Son

**Affiliations:** 1 Chung-Ang University Seoul Republic of Korea

**Keywords:** deep learning, epigenomics, genomics, lipidomics, machine learning, metabolomics, ML, multiomics, proteomics, risk stratification, stroke, transcriptomics

## Abstract

**Background:**

Stroke is a complex, multidimensional disorder influenced by interacting inflammatory, immune, coagulation, endothelial, and metabolic pathways. Single-omics approaches seldom capture this complexity, whereas multiomics techniques provide complementary insights but generate high-dimensional and correlated feature spaces. Machine learning (ML) offers strategies to manage these challenges; however, the predictive accuracy and reproducibility of multiomics-based ML models for stroke remain poorly characterized.

**Objective:**

This review aimed to conduct a systematic evaluation of ML models using multiomics data for stroke risk stratification and comprehensive patterns in discriminatory performance, integration strategies, and validation and reporting practices to inform future methodological development.

**Methods:**

We conducted a comprehensive literature search following PRISMA (Preferred Reporting Items for Systematic Reviews and Meta-Analyses) 2020 recommendations. Studies published from January 2000 to July 2025 were identified across 9 databases, including PubMed, MEDLINE Ultimate, EMBASE, CINAHL, Web of Science, Scopus, Cochrane CENTRAL, ACM Digital Library, and IEEE Xplore. Eligible studies included adults with ischemic, hemorrhagic, or unspecified stroke as the prediction target; applied at least 2 omics layers; and reported ML performance metrics. Risk of bias was assessed using the Prediction Model Risk of Bias Assessment Tool, while reporting quality was evaluated using Minimum Information for Medical AI Reporting. The primary outcome was the area under the receiver operating characteristic curve.

**Results:**

A total of 7 studies (n=40,274) published between 2022 and 2025 fulfilled the inclusion criteria. All studies combined 2 omics layers, most often using middle-level integration with dyads such as metabolomics-proteomics and metabolomics-lipidomics. Supervised ML algorithms across studies included support vector machines, tree-based ensembles, generalized linear models, and deep learning architectures. Three studies reported external validation of the integrated multiomics model, while 1 study conducted only an external assessment of a single marker rather than validation of the integrated model. Three studies reported an assessment of calibration, and clinically prespecified operating points were rarely described. Reported areas under the receiver operating characteristic curve varied by prediction task, ranging from 0.75 to 0.96 for acute diagnosis models and from 0.75 to 0.97 for onset risk prediction models; the highest externally validated performance was achieved by a support vector machine trained on a metabolomics-proteomics dyad in mixed stroke types (ischemic and hemorrhagic).

**Conclusions:**

Multiomics ML models showed high apparent discrimination for stroke risk stratification, but current evidence remains methodologically limited. Small sample sizes, heterogeneous designs, and incomplete reporting currently hinder the reproducibility and generalizability of multiomics ML models for stroke risk prediction. To advance the field, future studies should adopt leakage-resistant evaluation frameworks, conduct site-specific external validations, and benchmark against both single-omics and clinical baselines to demonstrate incremental value. Well-designed, transparently reported investigations will be essential to move multiomics ML models from exploratory promise toward clinically actionable tools in precision stroke care.

## Introduction

Stroke is defined by acute neurological deficits resulting from vascular brain injury [1,2]. Globally, this condition is responsible for about 6.6 million deaths annually, making it the second leading cause of mortality. Its contribution to disability-adjusted life years is anticipated to rise considerably by 2050 [3]. As stroke is associated with long-term functional impairment, cognitive decline, and a high risk of recurrence, early risk stratification remains critical [3-6]. Multiple layers of interacting risk factors shape the occurrence and progression of stroke [7]. The prediction of stroke occurrence and clinical course is influenced by a number of factors at the molecular level, including genetic predisposition, epigenetic alterations, chronic inflammatory responses, and metabolic abnormalities [8-13]. Although this multilayered pathophysiology helps explain heterogeneity in etiological classification, its complexity makes it difficult to capture fully using only clinical variables or a single biomarker [13-17].

Recently, multiomics approaches have been used to characterize cross-layer relationships—genetic, epigenetic, transcriptomic, proteomic, metabolomic, and lipidomic—within integrated analytic frameworks [13,15,18,19]. This reflects the inherently multilayered biology of stroke, in which inflammatory, immune, coagulation, and metabolic axes operate concurrently [17,20]. Prior evidence indicates that multiomics strategies may outperform single-omics approaches in clinical applications, including stroke subtype classification, risk prediction, and acute diagnosis [19,21]. Simultaneously, machine learning (ML) has proven particularly effective for analyzing multiomics data, which are marked by high dimensionality and nonlinearity [22,23]. ML enables the flexible integration of clinical variables with omics layers, supports reproducible analytic pipelines, and strengthens the performance and generalizability of prediction models [23,24]. Accordingly, there is increasing interest in developing precision ML models that leverage multidimensional biological data.

Although studies applying multiomics and ML to stroke have increased rapidly, substantial heterogeneity is evident across investigations in terms of the types of omics incorporated, integration strategies, prediction end points, predictor variables, evaluation metrics, disease spectra, validation methods, and sample sizes [25,26]. This variability limits cross-model comparability and complicates assessments of clinical applicability [27]. Nevertheless, comprehensive comparative and quantitative evaluations of the relative effectiveness of these approaches for stroke prediction remain scarce, and no systematic review synthesizing this evidence has been published to date.

This study aimed to perform a systematic analysis of ML-based investigations using multiomics data for stroke risk stratification and identify comprehensive performance patterns across the included studies to inform future methodological development.

## Methods

### Study Design

This review was conducted and reported in line with the PRISMA (Preferred Reporting Items for Systematic Reviews and Meta-Analyses) 2020 recommendations (Multimedia Appendix 1) [28]. Prior to conducting the review, the study protocol was prospectively registered in the PROSPERO (International Prospective Register of Systematic Reviews; registration number CRD420251089823).

### Data Sources and Search Strategy

A systematic search was conducted in PubMed, MEDLINE Ultimate (EBSCOhost), EMBASE, CINAHL, Web of Science, Scopus, Cochrane CENTRAL, ACM Digital Library, and IEEE Xplore for records published between January 1, 2000, and July 31, 2025. The start date was chosen a priori to capture early applications of omics-based prediction and to avoid arbitrarily excluding potentially relevant studies at the interface of omics and ML. Because multiomics and ML terms are inconsistently indexed across databases, we deliberately adopted a highly sensitive search strategy using multiple overlapping biomedical and multidisciplinary databases. Search strategies combined controlled vocabulary and free-text terms related to stroke, omics, and ML. Controlled vocabularies included Medical Subject Headings, Emtree, and CINAHL Headings; database-specific syntax and Boolean operators were adapted to each platform. Full search details for all databases are available in Multimedia Appendix 2. Gray literature—major preprint servers, including medRxiv, bioRxiv, and Google Scholar—were searched to check for any potentially missed or in-press studies; however, no additional records meeting the inclusion criteria were identified (final search date: July 31, 2025).

### Inclusion and Exclusion Criteria

Study eligibility was determined through the PICO (participants, interventions, comparisons, and outcomes) framework and is summarized in Table 1. Studies were included if they (1) enrolled adults (aged ≥18 years) with stroke or at risk of stroke (ischemic, hemorrhagic, or unspecified), (2) used multiomics data to develop ML models, and (3) reported model performance for at least 1 stroke-related prediction outcome. In this review, stroke-related outcomes included incident stroke risk, acute stroke diagnosis among patients with suspected stroke, and etiological subtype classification. In addition, we adopted a pragmatic, prediction-oriented definition of ML. Eligible models included supervised algorithms that used multiomics data as input to predict the prespecified outcomes. Studies were excluded if they targeted combined conditions rather than stroke alone, did not aim to predict stroke, substituted nonomics data for molecular omics, applied only omics or only ML without integration, or evaluated treatment effects. Conventional regression analyses conducted exclusively for explanatory or associative purposes without a specified predictive task were excluded, as they were not considered ML. The publication period (January 1, 2000, to July 31, 2025) was chosen to capture the evolution of multiomics- and ML-based stroke risk prediction from its early emergence to the most recent developments.

**Table 1:** Eligibility criteria for the systematic review using the PICO (participant, intervention, comparison, and outcomes) framework.

Item	Inclusion criteria	Exclusion criteria
Participants	Studies enrolling adults (aged ≥18 years) with stroke or at risk of stroke (ischemic, hemorrhagic, or unspecified)	Studies involving children or adolescents (aged <18 years) Studies restricted to demographic-only strata (eg, only men) Studies focusing on combined conditions (eg, stroke with dementia or atrial fibrillation) rather than stroke alone
Intervention	Studies using multiomics data (genomics, transcriptomics, proteomics, metabolomics, etc) to develop ML^a^ models for stroke prediction	Studies that do not aim to predict stroke Studies applying only omics or only ML, without integration of both Studies evaluating treatment effects (eg, drugs, procedures, or interventions) Studies using nonomics data (eg, imaging data such as CTb, MRIc, EEGd, or ultrasound) instead of molecular omics for stroke prediction
Comparison	No restrictions based on comparison	No restrictions based on comparison
Outcomes	Studies reporting prediction performance of ML models for stroke-related outcomes	Studies that do not report prediction performance
Language	No restrictions based on language	No restrictions based on language
Publication period	Studies published from January 1, 2000, to July 31, 2025	Studies published before January 1, 2000
Study designs	Original research articles, including observational studies, retrospective or prospective cohort studies, secondary data analyses, or basic science studies	Reviews, meta-analyses, protocols, editorials, or nonhuman studies Feasibility-only pilot studies without full model development or performance reporting
Publication types	Full published original articles in peer-reviewed journals	Abstract-only records, letters, withdrawn papers, or documents without full text

^a^ML: machine learning.

^b^CT: computed tomography.

^c^MRI: magnetic resonance imaging.

^d^EEG: electroencephalogram.

### Study Selection, Data Extraction, and Synthesis

Records retrieved from all sources were imported into EndNote 21 (Clarivate Plc) for deduplication and screening management, with tracking and extraction maintained in Microsoft Excel 2021 (Microsoft Corp). Titles and abstracts were independently screened in duplicate by 2 reviewers (HYY and HS) based on predefined eligibility criteria. Full texts of potentially eligible reports and risk of bias were also assessed independently in duplicate by 2 reviewers (HYY and HS). Disagreements between reviewers were resolved through discussion and, when necessary, adjudicated by a third reviewer (Y-JS). Data were extracted independently by 2 authors (HYY and HS) using a standardized piloted form and cross-verified by a third author (Y-JS). Extracted items included study title, authors, publication year, country, multiomics studied, sample size, data source, omics type, stroke type, prediction task, omics analytic methods, ML algorithms, outcome metrics and summary results, and validation details (internal/external and strategy). Model performance metrics were extracted and summarized descriptively. The primary measure of discrimination was the area under the receiver operating characteristic curve (AUC) with 95% CIs.

When discrimination was reported using Harrell C statistic for a binary outcome rather than a receiver operating characteristic (ROC)–based AUC, we treated the C statistics as numerically equivalent to the AUC for the purpose of pooling [29,30]. Publication bias is typically assessed when at least 10 studies are available; because fewer studies met the inclusion criteria in this review, publication bias was not formally evaluated, and no funnel plots were generated [31].

A meta-analysis was not performed because the limited number of eligible studies and the substantial between-study heterogeneity precluded meaningful quantitative synthesis. The included studies varied markedly in study design (retrospective vs prospective), sample size, and data sources (single-center cohorts, community-based datasets, and biobank data). In addition, the prediction tasks differed considerably, encompassing stroke onset risk prediction, acute diagnosis, and subtype classification. Given this conceptual, methodological, and statistical heterogeneity, quantitative pooling of effect estimates was judged inappropriate. Instead, we conducted a structured narrative synthesis to systematically summarize study characteristics, omics layers evaluated, ML approaches applied, and clinical prediction targets.

### Quality and Risk of Bias Assessment

Quality appraisal and risk of bias assessment were conducted using 2 frameworks suited to prediction modeling and medical artificial intelligence (AI) reporting: the Prediction Model Risk of Bias Assessment Tool (PROBAST) [32], and the Minimum Information for Medical AI Reporting (MINIMAR) [33]. PROBAST was used for the primary risk of bias classification, and was applied across the participants, predictors, outcomes, and analysis domains, with judgments at both the domain and overall levels categorized as low risk, high risk, or unclear [32]. MINIMAR was adopted as the checklist for AI and ML studies, as it represents an expert-developed framework suited to evaluating reporting quality in omics-based ML research, particularly in early translational health care contexts [33,34]. Each item was coded Y (adequate), P (partial), N (not reported), or X (not applicable), and compliance (%) was calculated as the proportion of Y among applicable items.

### Ethical Considerations

The institutional review board of Chung-Ang University (number 1041078-20250715-HR-225) approved the study protocol.

## Results

### Literature Search Results

The database search across 9 sources yielded 2960 records. Of these, 881 duplicates were excluded, leaving 2079 records to be screened. During title and abstract screening, 1413 records were excluded. Full texts were obtained for 666 reports, of which 530 were excluded as off-topic following full-text assessment.

**Figure 1:**
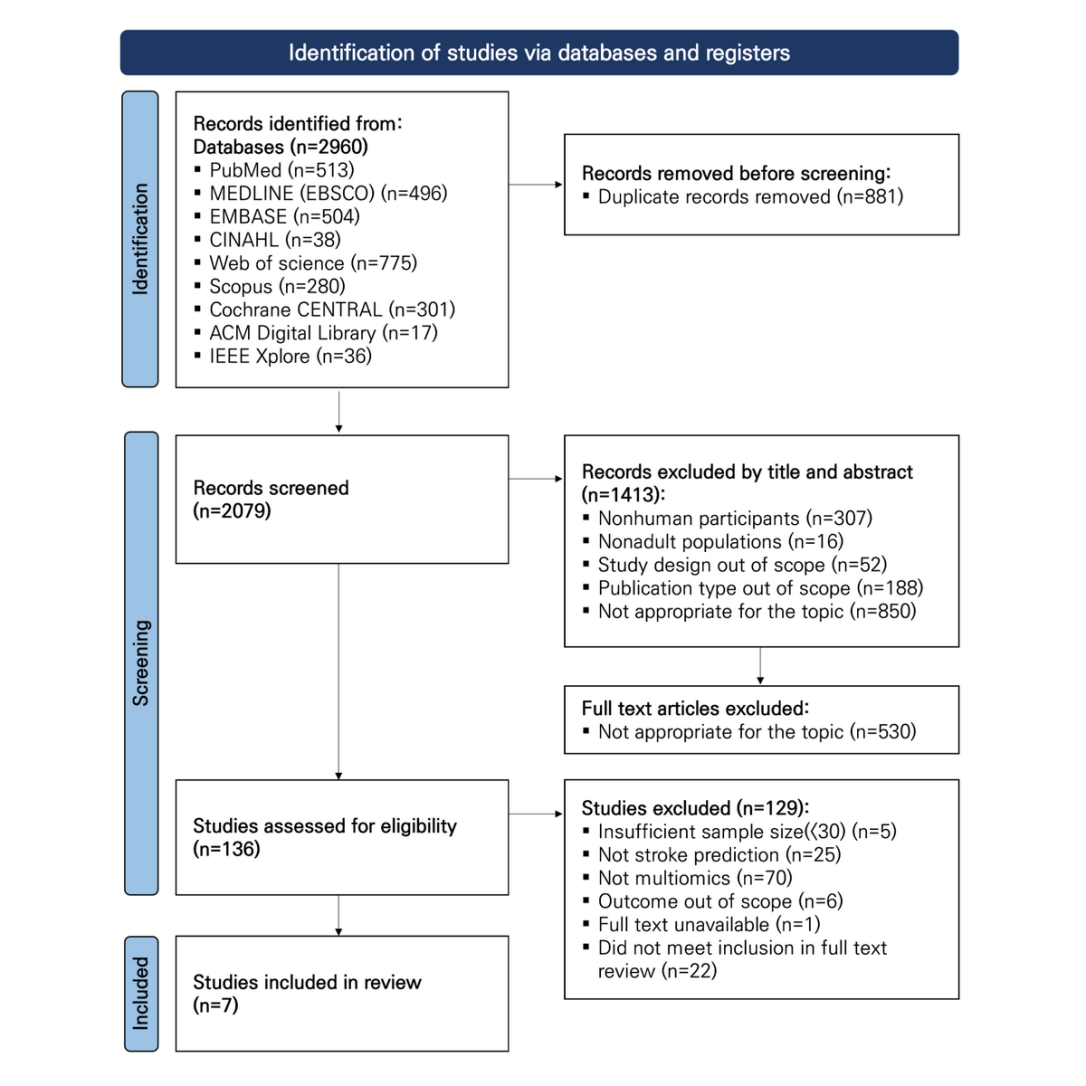
PRISMA flow diagram of study selection for systematic review of multiomics-based machine learning models for stroke prediction.

The remaining 136 studies underwent full eligibility evaluation. Of these, 129 were excluded for the following reasons: insufficient sample size (<30; n=5), not a stroke prediction study (n=25), not multiomics (n=70), outcome outside scope (n=6), full text unavailable (n=1), and failure to meet inclusion criteria on full-text review (n=22). A total of 7 studies were identified as meeting the inclusion criteria and were subsequently analyzed in this review (Figure 1) [35-41]. Gray literature searches yielded no additional records; therefore, the PRISMA flow diagram reflects only the results from database searches.

### Quality Assessment of the Included Studies

Study appraisals are summarized in Multimedia Appendix 3. On PROBAST, 4 studies were rated as low risk of bias [36,37,40,41], 2 studies as high risk [38,39], and 1 study as unclear overall [35]. Most concerns were concentrated in the analysis domain, whereas participants, predictors, and outcomes were generally judged to be at low risk. Applicability issues were minimal across the included studies. On MINIMAR, overall reporting compliance ranged from 11 of 21 items [35] to 17 of 21 items [41], with a median value of 14 of 21. Information on demographic characteristics (race, ethnicity, and socioeconomic status), intended users, data missingness, external validation, and data transparency was often insufficiently reported or entirely absent.

### Characteristics of the Included Studies

A total of 7 studies published between 2022 [38] and 2025 [36,41] were included in this review (Table 2). The studies represented 3 countries: China (n=4) [36-38,40], Spain (n=1) [39], and the United Kingdom (n=1; from England, Scotland, and Wales) [41]. Studies that used public repositories were classified as multinational [35,36]. Data sources were grouped into 3 categories: clinical cohorts recruited from hospitals or community health service centers (n=4) [37-40], public datasets (n=2) [35,41], and mixed sources (n=1) [36]. In the mixed-sources study [36], models were developed using gene expression omnibus datasets [42] and externally validated in independently recruited local clinical cohorts. Study designs were predominantly prospective (n=4) [37-39,41], with retrospective (n=2) [35,36], and mixed retrospective-prospective (n=1) [40]. Sample sizes varied widely, ranging from 30 participants in a hospital cohort [39] to 38,060 participants in a large public dataset [41].

Reported ML approaches included random forest, support vector machine (SVM), gradient boosting (extreme gradient boosting; adaptive boosting), generalized linear and logistic models, Bayesian logistic regression, the least absolute shrinkage and selection operator (LASSO), including the LASSO-penalized Cox proportional hazards model, and deep learning architectures such as the biological multilayer graph neural network. All studies integrated 2 omics layers, and the most frequent dyads were lipidomics-metabolomics (n=2) [37,38].

Regarding stroke type, 6 studies [35-39,41] specifically investigated ischemic stroke, whereas 1 study [40] included all stroke types. Prediction targets were grouped into categories: onset risk prediction (n=3) [35,40,41], acute diagnosis (n=2) [37,38], subtype classification (n=1) [39], and both reporting of diagnosis and subtype classification (n=1) [36].

**Table 2:** Characteristics of the included studies (N=7) of multiomics-based machine learning models for stroke prediction. The table summarizes key design features of each included study. Sample size (n) refers to the development cohort size for each study.

Study (year)	Country	Data source	Study design		Sample size, n	Machine learning algorithms	Omics types	Prediction task	
								Stroke type	Target
Liu et al [35] (2024)	Multinational	IEU^a^ and GEO^b^ database	Retrospective		103	RF^c^, SVM^d^, XGB^e^, and GLM^f^	Genomics and transcriptomics	Ischemic stroke	Onset risk prediction
Chen et al [36] (2025)	Multinational and China	GEO database, 2022-2023 single hospital		Retrospective	1785	RF and LASSO^g^	Transcriptomics and proteomics	Ischemic stroke	Acute diagnosis; subtype classification
Zhao et al [37] (2023)	China	2017-2019 CCPTP^h^		Prospective	90	RF and BLR^i^	Lipidomics and metabolomics	Ischemic stroke	Acute diagnosis
Ye et al [38] (2022)	China	Single hospital		Prospective	106	RF, SVM, and LR^j^	Lipidomics and metabolomics	Ischemic stroke	Acute diagnosis
Labarga et al [39] (2024)	Spain	2015-2016 single hospital		Prospective	30	BioMGNN^k^ and XGB	Transcriptomics and epigenomics	Ischemic stroke	Subtype classification
Zeng et al [40] (2024)	China	2010-2011 community health service centers, 2021-2023 single hospital		Prospective and retrospective	100	XGB, SVM, GNB^l^, RF, AdaBoost^m^, and L1-regularized LR	Metabolomics and proteomics	Stroke	Onset risk prediction
Gan et al [41] (2025)	England, Scotland, and Wales	UKB^n^		Prospective	38,060	LASSO-Cox^o^	Proteomics and genomics	Ischemic stroke	Onset risk prediction

^a^IEU: integrative epidemiology unit.

^b^GEO: gene expression omnibus.

^c^RF: random forest.

^d^SVM: support vector machine.

^e^XGB: extreme gradient boosting.

^f^GLM: generalized linear model.

^g^LASSO: least absolute shrinkage and selection operator regression.

^h^CCPTP: community comprehensive prevention and treatment project.

^i^BLR: binary logistic regression.

^j^LR: logistic regression.

^k^BioMGNN: biological multilayer graph neural network.

^l^GNB: Gaussian naïve bayes.

^m^AdaBoost: adaptive boosting.

^n^UKB: UK biobank.

^o^LASSO-Cox: least absolute shrinkage and selection operator-penalized Cox proportional hazards model.

### Omics Features and Integration Strategies in Multiomics Stroke Modeling

Table 3 summarizes the characteristics of omics data, their analytic roles, and the integration strategies applied in each study. Omics type lists all molecular layers included per study, and biospecimen records the biological source of each layer as reported. The analysis unit indicates the level of the final features entered into the model.

**Table 3:** Omics features and integration strategies for multiomics stroke modeling in the included studies (n=7).

Study (year) and omics data type			Biospecimen		Analysis unit		Top-ranked feature biomarkers		Role in pipeline		Feature selection method		Integration strategy	
**Liu et al** **[35] (2024)**														Middle integration
	Genomics	GWAS^a^ summary data		SNP^b^ to gene-level		*FURIN (OMIM 136950)*, *TOMM40 (OMIM 608061)*, *HDDC3 (NCBI Gene 374659)*, *ALDH2 (OMIM 100650), MAN2A2 (OMIM 600988)*		FS^c^, FP^d^, RS^e^		LD^f^ clumping with genome-wide significance and F-stat				
	Transcriptomics	Whole-blood mRNA^g^		Gene-level expression		*FURIN, TOMM40, HDDC3, ALDH2, MAN2A2*		FS, FP, VAL^h^		DEAGs^i^				
**Chen et al [36]** **(2025)**														Late integration
	Transcriptomics	Serum miRNA^j^, whole-blood mRNA		miRNA expression, gene-level mRNA expression		hsa-miR-3646, hsa-miR-4669, hsa-miR-4721, hsa-miR-504-3p, hsa-miR-8085, hsa-miR-3064-5p, hsa-miR-6736-5p, hsa-miR-6835-5p, hsa-miR-6793-5p		FS, FP		DEMs^k^, ML^l^ selection				
	Proteomics	Plasma protein		Protein level		ITGAM^m^		FS, VAL		Cross-omics prioritization, targeted validation				
**Zhao et al [37]** **(2023)**														Middle integration
	Lipidomics	Serum		LC-MS^n^ untargeted metabolite features		PE^o^ (18:0p/18:2), PE (16:0e/20:4), OAHFA^p^ (36:3), PE (16:0p/20:3), PE (18:1p/18:2)		FS, FP, VAL		PLS-DA^q^, ML selection				
	Metabolomics	Serum		LC-MS untargeted lipid species		4-hydroxyphenylpyruvic acid, cafestol		FS, FP, VAL		PLS-DA, ML selection				
**Ye et al [38]** **(2022)**														Middle integration
	Lipidomics	Serum		LC-MS untargeted metabolite features		PE (22:6/P-18:0), Cer^r^ 34:2, GlcCer^s^ (d18:0/18:0), DG^t^ 44:0, LysoPC^u^ (16:0), 22:6-OH^v^/LysoPC, TAG^w^ 58:7-FA^x^ 22:4		FS, FP		OPLS-DA^y^, receiver operating characteristic-based evaluation				
	Metabolomics	Serum		LC-MS untargeted lipid species, targeted lipid species		Taurine, oleoylcarnitine, creatinine		FS, FP		OPLS-DA, receiver operating characteristic-based evaluation				
**Labarga et al [39] (2024)**														Middle integration
	Transcriptomics	Whole-blood mRNA, miRNA, circRNA^z^		Gene-level expression, miRNA expression, circRNA expression		Not reported (high-dimensional omics features; candidate biomarkers mentioned but no explicit ranking provided)		FS, FP, VAL		GraphSAGE^aa^-based embedding, attention weight				
	Epigenomics	Whole-blood DNA methylation		CpG^ab^ β-values		Not reported (high-dimensional omics features; candidate biomarkers mentioned but no explicit ranking provided)		FS, FP, VAL		Probe-level quality control, GraphSAGE-based embedding, attention weight				
**Zeng et al [40] (** **2024)**														Early integration
	Metabolomics	Plasma		LC-MS untargeted metabolite features		Caprolactam		FS, FP, VAL		OPLS-DA, ML selection				
	Proteomics	Plasma		DIA^ac^ protein groups		C4BPA^ad^, COL15A1^ae^, HBB^af^		FS, FP, VAL		DEPs^ag^, ML selection, cross-model intersection				
**Gan et al [41]** **(2025)**														Late integration
	Proteomics	Plasma		Antibody-based proteomics analytes		17-protein score (GDF15^ah^, PLAUR^ai^, NT-proBNP^aj^, IGFBP4^ak^, BCAN^al^ +12 proteins)		FS, FP, RS, VAL		LASSO-Cox^am^, 17-protein score derived				
	Genomics	Whole-blood DNA genotypes		PRS^an^		Not reported		FP, RS, VAL		Precomputed PRS				

^a^GWAS: genome-wide association study.

^b^SNP: single nucleotide polymorphism.

^c^FS: feature selection.

^d^FP: final predictors.

^e^RS: risk score.

^f^LD: linkage disequilibrium.

^g^mRNA: messenger RNA.

^h^VAL: validation.

^i^DEAGs: differentially expressed associated genes.

^j^miRNA: microRNA, microRNA of human origin.

^k^DEMs: differentially expressed miRNAs.

^l^ML: machine learning.

^m^ITGAM: integrin subunit alpha M.

^n^LC-MS: liquid chromatography-mass spectrometry.

^o^PE: phosphatidylethanolamine.

^p^OAHFA: (O-acyl)-1-hydroxy fatty acid.

^q^PLS-DA: partial least squares-discriminant analysis.

^r^Cer: ceramide.

^s^GlcCer: glucosylceramide.

^t^DG: diacylglycerol.

^u^LysoPC: lysophosphatidylcholine.

^v^OH: hydroxyl group.

^w^TAG: triacylglycerol.

^x^FA: fatty acid.

^y^OPLS-DA: orthogonal partial least squares-discriminant analysis.

^z^circRNA: circular RNA.

^aa^GraphSAGE: graph sample and aggregate.

^ab^CpG: cytosine phosphate guanine.

^ac^DIA: data-independent acquisition.

^ad^C4BPA: complement component 4 binding protein alpha.

^ae^COL15A1: collagen type XV alpha 1 chain.

^af^HBB: hemoglobin subunit beta.

^ag^DEPs: differentially expressed proteins.

^ah^GDF15: growth differentiation factor 15.

^ai^PLAUR: plasminogen activator, urokinase receptor.

^aj^NT-proBNP: N-terminal pro b-type natriuretic peptide.

^ak^IGFBP4: insulin-like growth factor binding protein 4.

^al^BCAN: brevican.

^am^LASSO-Cox: least absolute shrinkage and selection operator-penalized Cox proportional hazards model.

^an^PRS: polygenic risk score.

Top-ranked feature biomarkers represent the features or panels identified by authors as having the highest contributions. Role in pipeline indicates how each biomarker was used and is coded as feature selection, final predictors, validation, or risk score. Multiple codes may apply when roles differ across study phases. The feature selection method summarizes the dominant selector applied before model fitting. Integration method classifies how multiomics layers were combined, following established taxonomies [43,44]: early integration (simple concatenation of preprocessed omics into a single matrix for a downstream learner), middle integration (a joint latent representation learned directly across omics followed by modeling), and late integration (separate omics-specific models or scores combined at the decision or score level).

Across the 7 included studies, multiomics inputs comprised transcriptomics (n=3) [35,36,39], proteomics (n=3) [36,40,41], metabolomics (n=3) [37,38,40], lipidomics (n=2) [37,38], epigenomics (n=1) [39], and genomics (n=2) [35,41] (Table 3). Biospecimens were predominantly blood derived—whole blood for messenger RNA, circular RNA expression, and DNA-methylation; serum or whole blood for micro RNA (miRNA) profiling; and serum or plasma for protein panels and liquid chromatography–mass spectrometry (LC-MS)–based metabolite and lipid features. Genomic data were sourced from genome-wide association study summary statistics in 1 study [35] and from whole blood DNA genotypes in another [41].

Most studies (n=6) reported top-ranked biomarkers or panels. By contrast, 1 study [39] used deep learning–based representation learning to process high-dimensional transcriptomic and epigenomic inputs directly as raw features, subsequently deriving multiomics embeddings from the full feature space. Consequently, no ranked lists of biomarkers were generated in this study. Regarding analytic roles, omics variables were consistently used for feature selection and as final predictors. A dedicated validation step—internal and/or external—was described in 5 studies [36-40], while a risk score stage was incorporated in 2 studies [35,41].

Given the heterogeneity of feature selection approaches, Table 3 provides a concise method label for each study. Regarding integration strategies, most studies used middle-level integration (n=4) [35,37-39], while late-level integration was reported in 2 studies [36,41] and early-level integration in 1 study [40]. Middle integration was implemented in three main forms: (1) cross-omics filtering, in which genome-wide association study signals were intersected with transcriptomic differentially expressed genes to define the feature set for ML; (2) projection-based selection, where lipidomic and metabolomic data were reduced to compact multiomics panels; and (3) graph-based joint embeddings. Late integration was used in two distinct ways: (1) validation-stage fusion, in which transcriptomic discoveries were linked with an independent proteomic readout to confirm cross-omics consistency; and (2) score-level fusion, where a protein risk score and a polygenic risk score were jointly incorporated into prediction models.

A study [40] used a mixed stroke end point combining ischemic and hemorrhagic stroke. Because this outcome definition merges etiologically distinct conditions, the identified features are interpreted as mixed-population signals rather than subtype-specific molecular biomarkers. Methodologically, the study performed early integration by analysis-specific quality control and univariate screening, followed by concatenation of the retained features into a single matrix for common selection (LASSO) and modeling (eg, SVM).

### Model Performance of Risk Prediction for Stroke

Predictive performance is summarized in Table 4. Discrimination was characterized primarily using AUC with 95% CIs; when available, sensitivity, specificity, validation method, and calibration details were also extracted. The representative performance shown for each study does not necessarily correspond to the numerically highest AUC. Primary estimates were selected to align with the objectives of this review. Multiomics integration and the use of clinically comparable control groups were prioritized as key criteria, and external validation results were adopted whenever available. If external validation was not reported, the best internally validated AUC was recorded. For Gan et al [41], the proteomics plus polygenic risk score specification was designated as the primary estimate because it most reflected multiomics integration, consistent with the inclusion criteria of this review. Further details are provided in the footnotes of Table 4.

**Table 4:** Predictive performance of multiomics-based machine learning models for stroke in adult population (N=7). For the primary model selected from each study, the table summarizes omics final predictors, validation approach, and key performance metrics. When external validation was reported, “n” indicates the sample size of the external validation cohort, reported separately from the development cohort.

Study (year)	Final predictors, n	Cross-validation	External validation	Calibration	Machine learning model performance				
					Model	AUC^a^ (95% CI)	Sensitivity		Specificity
Liu et al [35] (2024)	5	Not reported	Yes (n=12)	Yes^b^	GLM^c^	1.000 (1.000-1.000)	Not reported	Not reported	
Chen et al [36] (2025)	1^d^	10-fold	Partial (n=44)	Not reported	LASSO+RF^e^	0.750 (0.601-0.899)	0.567		0.857
Zhao et al [37] (2023)	7	7-fold	No	Yes	RF+LR^f^	0.837^g^ (0.732-0.942)	0.897		0.733
Ye et al [38] (2022)	10	Not reported	No	Not reported	SVM^h^	0.963 (0.884-1.000)	Not reported		0.917
Labarga et al [39] (2024)	High-dimensional omics features	5-fold	No	Not reported	BioMGNN^i^	0.950 (not reported)	0.950		Not reported
Zeng et al [40] (2024)	4	10-fold	Yes (n=44)	Yes	SVM	0.973 (0.921-0.999)	0.864		1.000
Gan et al [41] (2025)	2^j^	10-fold	Yes (n=4970)	Not reported	LASSO-Cox^k^	0.748^l^ (0.707-0.789)	Not reported	Not reported	

^a^AUC: area under the receiver operating characteristic curve.

^b^Calibration was reported in the Methods, but no plot or statistic was presented.

^c^GLM: generalized linear model.

^d^External AUC from integrin alpha M (ITGAM) measured in an independent clinical cohort.

^e^LASSO+RF: least absolute shrinkage and selection operator regression–based feature selection by random forest as the final classifier.

^f^RF+LR: random forest feature selection followed by logistic regression as the final classifier.

^g^Value shown is hypertensive ischemic stroke versus healthy controls.

^h^SVM: support vector machine.

^i^BioMGNN: biological multilayer graph neural network.

^j^Score-level fusion of proteomic and genetic risk scores.

^k^LASSO-Cox: least absolute shrinkage and selection operator-penalized Cox proportional hazards model.

^l^Harrell C statistic for a boundary outcome, treated as numerically equivalent to AUC for quantitative synthesis. The estimate corresponds to protein risk score + polygenic risk score (PRS) to reflect multiomics integration.

Final predictors in Table 4 refer to the predictors actually entered into the final model (eg, selected feature panel, single markers, or score-level inputs), excluding clinical covariates. Across studies, most models relied on compact omics-derived inputs, while 1 study [39] analyzed high-dimensional feature sets. For late-level integration, the final predictors correspond to the score-level [41] or marker-level [36] inputs entered into the model, rather than the underlying omics features used to construct them. Ten-fold cross-validation was the most commonly applied procedure (n=3) [36,40,41], while 7-fold [37] and 5-fold [39] schemes were also reported. Several studies [35,38] did not specify the resampling method. The algorithms most frequently applied were SVM and random forest, often coupled with LASSO for feature selection. Additional approaches included generalized linear models, the LASSO-penalized Cox proportional hazards model, and deep learning architectures such as a biological multilayer graph neural network.

A total of 3 studies [35,40,41] reported external validation of the integrated multiomics model, whereas Chen et al [36] reported an external assessment limited to a single biomarker, integrin alpha M. Three studies [35,37,40] reported calibration assessment. Although Liu et al [35] stated that calibration curves were generated, no plot or statistic was presented. Among externally validated models—excluding 1 boundary estimate (AUC=1.00) [35]—AUC values ranged from 0.75 [41] to 0.97 [40], both of which were onset risk prediction models. The only acute diagnosis model with external validation was Chen et al [36] (AUC=0.75); however, the external validation was conducted as a cross-omics single-marker assessment—integrin alpha M measured at the proteomic level—rather than validation of an integrated multiomics model. When all reported AUCs were stratified by clinical task regardless of validation type, acute diagnosis models reported AUCs ranging from 0.75 [36] to 0.96 [38], whereas onset risk prediction models reported AUCs ranging from 0.75 [41] to 0.97 [40]. Notably, the SVM model in Zeng et al [40] achieved the highest externally validated discrimination (AUC=0.97) using a metabolomics-proteomics combination. However, because this estimate was derived from a mixed stroke end point, it does not reflect performance for a specific stroke subtype. Where reported, sensitivity and specificity at the chosen decision thresholds are summarized in Table 4. A total of 5 studies [36-40] reported sensitivity and/or specificity, and only 3 studies [36,37,40] reported both metrics at prespecified decision thresholds. Because reporting was incomplete and thresholds and reporting formats were heterogeneous across studies, attempts at separate quantitative synthesis of sensitivity or specificity were not conducted. Reporting of model performance and validation procedures varies across studies, which limited comparability and may have contributed to optimistic estimates. Only 1 study [41] evaluated model performance against standard clinical predictors. In Gan et al [41], the multiomics model had a C statistic of 0.765 in internal validation and 0.748 in external validation, whereas the clinical predictor model showed corresponding values of 0.753 and 0.754, respectively.

## Discussion

### Overview

This systematic review synthesized 7 studies of multiomics-based ML models for stroke-related prediction tasks. To our knowledge, it is one of the first reviews to focus specifically on multiomics ML in the context of stroke risk stratification. Although many of the included models reported apparently high discrimination, these findings arise from small, heterogeneous, and methodologically limited evidence base characterized by high-dimensional omics inputs, limited external validation, and inconsistent reporting. Accordingly, the primary contribution of this review is not to demonstrate that current multiomics models are ready for clinically meaningful or generalizable accuracy, but rather to show that the current evidence base is methodologically limited, prone to performance inflation, and in need of substantially more rigorous and transparent evaluation.

### Discrimination and Methodological Quality in Multiomics-Based Machine Learning Stroke Prediction

On face value, most included multiomics ML models reported high apparent discrimination, particularly when compact biomarker panels were constructed from biologically coherent omics dyads. However, these apparently strong AUCs must be interpreted with extreme caution. Across studies, small-to-moderate sample sizes, high-dimensional inputs, and optimistic validation strategies created ideal conditions for severe overfitting and data leakage. Within this constrained context, some technical patterns were still observable. For example, a metabolomics-proteomics SVM reported an externally validated AUC of 0.97 with a boundary CI (95% CI 0.92-1.00) [40]. This finding is often interpreted as evidence that compact and mechanically coherent biomarker panels can match or exceed more complex multilayered approaches [45]. At the algorithmic level, the prominence of SVM in the best-performing models is also broadly consistent with previous meta-analytic work in cardiovascular ML, in which SVM achieved slightly higher pooled AUCs for stroke outcomes than boosting algorithms or convolutional neural networks [46]. In the specific metabolomics-proteomics SVM model in this review, the combination of a modest sample size and almost upper-limit confidence bound suggests that residual overfitting and optimism remain likely, even with external testing.

A deep learning model applied to transcriptomic-epigenomic matrix also reported an AUC approaching 0.95 [39]; however, this estimate was generated in a cohort of only 30 participants in a pronounced “p≫n” biomarker-discovery setting. This high AUC is therefore best viewed as a methodological artifact rather than a valid predictive performance. Without leakage-robust pipelines and adequately powered external validation, this result should be considered exploratory and should not be used to claim strong discrimination. In this context, the combination of high-capacity architecture, high-dimensional feature space, and limited effective sample size makes severe overfitting and information leakage not merely possible but statistically expected. In addition, the study did not report specificity at prespecified decision thresholds, which makes it difficult to relate the reported sensitivity to false-positive rates and limits the clinical interpretability of the findings [47]. The generalized linear model–based study reported an AUC of 1.00 [35]. Given the small sample size and high-dimensional feature space, this boundary estimate is more appropriately interpreted as an optimistic methodological artifact rather than valid or generalizable predictive performance [48,49]. In addition, calibration was described but not shown; the absence of explicit plots or statistics limits contextual interpretation of the apparent perfect separation. Taken together, these findings reinforce that near-ceiling discrimination estimates in this review are not reliable indicators of clinical validity. Overall, the reported performance should be viewed as preliminary and potentially optimistic given the methodological constraints of the included studies.

### Observed Performance Patterns by Integration Strategy and Omics Layers

The performance of multiomics ML models appears to be influenced by both the integration strategy and the specific omics layers used. Within this small and heterogeneous set of studies, most applied middle-level integration, which offered a pragmatic balance for limited sample sizes and heterogeneous data platforms by generating joint feature representations prior to classification [50,51]. Early-level integration, while more vulnerable to dimensionality challenges, produced strong results when combined with stringent feature selection and relatively careful validation [52], as demonstrated in one of the higher-performing studies in this review. By contrast, late-level integration, implemented either as score-level fusion or cross-omics validation, reported more modest AUC values (0.75) [36,41]. Notably, Gan et al [41] used score-level fusion by combining a proteomic risk score with a polygenic risk score, rather than feature-level integration. This formulation may be less able to capture complex interomics relations [44,53]. Accordingly, several methodological studies have suggested that late-level integration may underperform because it cannot fully capture synergistic cross-layer interactions [51,52]. In this review, however, these differences cannot be attributed to integration strategy alone, because the late-integration studies also differed in prediction task, sample size, and model specification. Thus, the apparent underperformance of late-level integration in this review should be regarded as a hypothesis-generating observation rather than a general conclusion about the inherent limitation of late-integration approaches.

Beyond integration strategies, the selection of omics layers fundamentally shaped predictive utility. Consistent with prior research, transcriptomic-epigenomic pairs were generally used to represent relatively stable regulatory states, whereas lipidomic-metabolomic dyads were used to capture pathway dynamics and acute physiological responses [54-56]. Genomic data mostly contributed a predispositional context rather than serving as direct predictive variables. Additionally, the definition of the analysis unit—for example, gene-level expression, CpG probe values, or LC-MS features—determined the imputation burden, multiplicity, and scope of feasible feature selection methods, thereby influencing both model performance and reproducibility [55,57,58]. Collectively, these observations underscore that methodological design choices in omics integration and feature representation are not simply technical decisions but pivotal determinants of translational potential.

### Cross-Study Heterogeneity and Barriers to Quantitative Synthesis

Across the included studies, differences in reported performance primarily reflected variations in study design and analytic choices rather than random fluctuation. Substantial heterogeneity was observed across study populations, prediction targets, stroke end points, and analytic units. In this context, the evidence was synthesized narratively rather than summarized as a single pooled effect size.

More specifically, formal pooling of AUCs was not undertaken because it would have required combining performance estimates derived from substantially different data structures and outcome definitions. With respect to the omics inputs, some studies modeled gene- or protein-level summaries, whereas others analyzed large sets of individual probes or untargeted LC-MS features, leading to marked differences in dimensionality and in the feature-selection and validation strategies that were feasible. At the outcome level, 1 study modeled composite “all-stroke” end points that combined ischemic and hemorrhagic events, whereas others focused exclusively on ischemic stroke. Because these subtypes have distinct pathophysiology and omics profiles, mixed end points limit subtype-specific interpretation and contribute to heterogeneity across studies. Consequently, similar AUC values may reflect different biological mechanisms, case mixes, and follow-up structures, and a single pooled estimate would provide limited additional clinical or methodological insight. Overall, this pattern of heterogeneity is consistent with an early, exploratory phase of multiomics ML research in stroke and highlights the need for more standardized designs and reporting.

Comparable patterns of methodological divergence have been described in broader reviews of ML-based prediction models, in which variation in datasets, predictors, analytic pipelines, and algorithms has constrained the interpretability of pooled accuracy estimates [46,59-61]. Our findings are consistent with this broader literature: the included studies are sufficiently mature for qualitative comparison but not yet harmonized to support robust quantitative synthesis.

### Implications for Practice and Future Research

For time-critical decisions in hyperacute stroke care, integrating current multiomics workflows into the minute-to-hour decision windows that govern acute management remains challenging. In particular, most acute diagnosis studies in this review relied on omics profiling that generally depends on centralized laboratory workflows (eg, LC-MS–based proteomic/metabolomic assays). Given current turnaround times, these approaches are unlikely to be clinically translatable within hyperacute treatment windows, limiting near-term clinical feasibility [62]. From a broader risk-stratification perspective, however, multiomics ML approaches may be better suited to longer time horizons, including identification of individuals at elevated future stroke risk, refinement of etiologic classification after the acute phase, and delineation of biologically homogeneous patient subgroups that are not apparent from clinical variables or imaging alone [63,64]. Any future role in acute pathways would require both stronger predictive evidence and assay platforms with substantially shorter turnaround times and lower costs.

Within this context, the current evidence does not yet provide a sufficiently robust foundation for routine implementation of multiomics-based ML models for stroke risk stratification. These models are more accurately regarded as exploratory prototypes, demonstrating potential capabilities within an optimized analytical framework. Compact, blood-derived biomarker panels constructed from biologically coherent omics dyads may offer a plausible route for future clinical application, provided that their performance can be reliably replicated and calibrated in adequately powered, prospectively designed studies using appropriate analytic platforms [65,66].

From a methodological perspective, the studies reviewed highlight critical domains requiring standardization. Repository-based cohorts provide statistical power and facilitate discovery, but they may obscure preanalytical variability and heterogeneity in investigator judgment [67,68]. By contrast, locally recruited cohorts enable clinically focused hypotheses but often suffer from limited sample sizes [69]. Furthermore, the choice of analytic unit—whether gene, CpG probe, protein, or LC-MS feature level—emerged as a critical determinant of which feature selection and integration techniques were feasible, thereby exerting a direct influence on predictive performance. Collectively, these observations underscore the imperative for harmonized analytic frameworks and transparent reporting standards to strengthen reproducibility and enable meaningful cross-study comparability.

Prospective, multisite validation of compact and biologically coherent panels across diverse populations should be a priority. Such studies must incorporate leakage-robust validation pipelines, systematically report calibration and decision-curve analyses, clinically relevant operating points (including sensitivity and specificity at prespecified thresholds), and benchmark performance against both single-omics models and established clinical baselines to quantify true incremental value [70,71]. In future work, both primary studies and evidence syntheses should avoid pooling ischemic and hemorrhagic stroke into undifferentiated “all-stroke” end points when quantifying model performance, given their distinct pathophysiological and multiomics profiles. These observations collectively support subtype-specific development and synthesis as a more appropriate strategy for future multiomics ML research in stroke. Expanding prediction targets to encompass hemorrhagic stroke, postevent prognostic outcomes, stroke recurrence in secondary prediction context, and health-economic evaluations will also be essential to define the broader clinical utility of multiomics ML approaches. With rigorous methodological safeguards and robust external validation, these models hold the potential to transition from exploratory promise to clinically actionable tools in precision cerebrovascular medicine.

### Limitations

This review has several limitations. First, although we initially planned to conduct a meta-analysis, we ultimately judged that conducting a meta-analysis would be inappropriate because the included studies differed markedly in sample size, data sources, validation strategies, and outcome definitions (eg, stroke onset risk prediction, acute diagnosis, and subtype classification); these discrepancies collectively precluded meaningful quantitative synthesis and robust pooled effect estimates. Second, external validation was conducted in only half of the studies, while calibration and clinically relevant operating points were reported in only a minority, limiting confidence in both reproducibility and clinical applicability. Third, most cohorts focused on ischemic stroke and blood-based assays, with only limited data for hemorrhagic or mixed all-stroke populations and alternative biospecimens, restricting generalizability and likely contributing further to clinical and biological heterogeneity. Fourth, reporting gaps—particularly concerning missing-data mechanisms, demographic characteristics, and preanalytical procedures—were frequent and constrained assessment of bias and transportability. Fifth, differences in prediction objectives and performance metrics complicated the cross-study interpretation. Furthermore, the selection of a single representative model in each study, while designed to ensure consistency, introduced subjectivity and may have underestimated the comprehensive range of model performance reported in individual studies. Regarding literature retrieval, the IEEE Xplore search relied on broad umbrella terms rather than a comprehensive set of omics layer–specific keywords, which may have reduced sensitivity for some technical records. Sixth, although we excluded studies with a sample size under 30, the included analyses involved small cohorts relative to the dimensionality of the omics data, making them highly susceptible to overfitting and inflated performance even when cross-validation was reported. Finally, methodological variability across biospecimens, analytic levels, and computational platforms further limited comparability across studies. Collectively, these limitations—including the small number of eligible studies, limited external validation, incomplete reporting, and methodological heterogeneity—underscore the need for standardized study designs, rigorous external validation, and transparent reporting practices to advance the field toward reproducible and clinically meaningful applications.

### Conclusion

This systematic review shows that multiomics-based ML models for stroke-related prediction tasks frequently reported high apparent discrimination, but almost exclusively in small, heterogeneous, and high-dimensional studies with limited external validation and incomplete reporting. Current performance estimates are therefore likely optimistic and are not sufficient to justify routine clinical use or to demonstrate superiority over single-omics or purely clinical models. Even the highest externally validated result should be viewed as a promising proof of concept rather than evidence of robust and clinically generalizable performance. Looking ahead, multiomics integration remains a plausible basis for more precise stroke risk stratification, but only if future models are developed and evaluated in adequately powered, prospectively planned, and transparently reported studies. Such work will require leakage-resistant validation frameworks, clear calibration and clinically relevant operating points, and systematic benchmarking against simpler alternatives. Under these conditions, multiomics-based ML models may progress from exploratory prototypes to robust tools that support earlier diagnosis and more tailored intervention in stroke care.

## Appendix

Multimedia Appendix 1 PRISMA checklist for systematic review.

Multimedia Appendix 2 Literature search strategy.

Multimedia Appendix 3 Critical appraisal and assessment of bias among studies.

## Abbreviations

AI: artificial intelligence
AUC: area under the receiver operating characteristic curve
LASSO: least absolute shrinkage and selection operator
LC-MS: liquid chromatography–mass spectrometry
MINIMAR: Minimum Information for Medical AI Reporting
ML: machine learning
PICO: participants, interventions, comparisons, and outcomes
PRISMA: Preferred Reporting Items for Systematic Reviews and Meta-Analyses
PROBAST: Prediction Model Risk of Bias Assessment Tool
PROSPERO: International Prospective Register of Systematic Reviews
ROC: receiver operating characteristic
SVM: support vector machine

## Data Availability

The data underlying the findings of this study are available from the corresponding author upon reasonable request.
