# Supplementary material for: Machine Learning for Predicting Stroke Risk Stratification Using Multiomics Data: Systematic Review
Source: J Med Internet Res. 2026 Feb 19;28:e85654. doi: 10.2196/85654 (PMC12963974; doi:10.2196/85654)
Supplement: Multimedia Appendix 2 [file jmir_v28i1e85654_app2.docx]

**Multimedia Appendix**. Literature search strategy

| **Database** | **Search No.** | **Search strategy** | **Articles retrieved** |
| --- | --- | --- | --- |
| PubMed | #1 | ("ischemic stroke"[Title/Abstract] OR "hemorrhagic stroke"[Title/Abstract] OR "brain Ischemia"[MeSH Terms] OR "brain Ischemia"[Title/Abstract] OR "cerebral ischemia"[Title/Abstract] OR "Stroke"[MeSH Terms] OR "brain infarct*"[Title/Abstract] OR "brain vascular accident*"[Title/Abstract] OR "cerebral stroke*"[Title/Abstract] OR "cerebral infarct*"[Title/Abstract] OR "cerebral accident*"[Title/Abstract] OR "ischemic stroke*"[Title/Abstract] OR "intracranial infarct*"[Title/Abstract] OR "lacunar stroke*"[Title/Abstract] OR "lacunar infarct*"[Title/Abstract] OR "stroke*"[Title/Abstract] OR "small vessel stroke*"[Title/Abstract] OR "Intracranial Arteriosclerosis"[MeSH Terms] OR "Cerebral Arterial Diseases"[MeSH Terms] OR "cerebral artery disease*"[Title/Abstract] OR "intracranial artery disease*"[Title/Abstract] OR "Intracranial Embolism and Thrombosis"[MeSH Terms] OR "brain embol*"[Title/Abstract] OR "cerebral embol*"[Title/Abstract] OR "cerebral thromb*"[Title/Abstract] OR "intracranial embol*"[Title/Abstract] OR "intracranial thromb*"[Title/Abstract] OR "cardioembolic stroke*"[Title/Abstract] OR "embolic stroke*"[Title/Abstract] OR "thrombotic stroke*"[Title/Abstract]) | 278,642 |
|  | #2 | ("multiomics"[MeSH Terms] OR "multi-omics"[Title/Abstract] OR "multiomics"[Title/Abstract] OR "multiomics"[MeSH Terms] OR "integrative omics"[Title/Abstract] OR "Genomics"[MeSH Terms] OR "Transcriptome"[MeSH Terms] OR "transcriptomics"[Title/Abstract] OR "transcriptome profil*"[Title/Abstract] OR "Proteomics"[MeSH Terms] OR "Proteomics"[Title/Abstract] OR "Proteome"[MeSH Terms] OR "Metabolomics"[MeSH Terms] OR "Metabolome"[MeSH Terms] OR "Metabolomics"[Title/Abstract] OR "bioinformatic analys*"[Title/Abstract] OR "whole genome"[Title/Abstract] OR "whole exome"[Title/Abstract] OR "genome-wide"[Title/Abstract] OR "RNAseq"[Title/Abstract] OR "RNA-seq"[Title/Abstract] OR "microarray"[Title/Abstract] OR "transcriptom*"[Title/Abstract] OR "genomic*"[Title/Abstract] OR "epigenomic*"[Title/Abstract] OR "proteomic*"[Title/Abstract] OR "metabolomic*"[Title/Abstract] OR "omic*"[Title/Abstract] OR "biomarker*"[Title/Abstract] OR "bioinformatics"[Title/Abstract]) | 820,498 |
|  | #3 | ("machine learning"[MeSH Terms] OR "machine learning"[Title/Abstract] OR "artificial intelligence"[Title/Abstract] OR "deep learning"[Title/Abstract] OR "transfer learning"[Title/Abstract] OR "support vector machine"[Title/Abstract] OR "XGBoost"[Title/Abstract] OR "random forest"[Title/Abstract] OR "gradient boosting machine"[Title/Abstract] OR "ensemble learning"[Title/Abstract] OR "neural network"[Title/Abstract] OR "neural networks"[Title/Abstract] OR "multilayer perceptron"[Title/Abstract] OR "k nearest neighbor"[Title/Abstract] OR "Adaboost"[Title/Abstract] OR "ResNet"[Title/Abstract] OR "decision tree"[Title/Abstract] OR "bayesian network"[Title/Abstract] OR "naive bayesian"[Title/Abstract] OR "risk model"[Title/Abstract] OR "prediction model"[Title/Abstract] OR "nomogram"[Title/Abstract]) | 216,314 |
|  |  | Filters: Full text, Humans, from 2000/1/1 – to 2025/07/31 |  |
|  |  | #1 AND #2 AND #3 | **513** |
| MEDLINE Ultimate  (EBSCOhost) | #1 | ( MH "brain ischemia" OR MH "stroke" OR MH "intracranial arteriosclerosis" OR MH "cerebral arterial diseases" OR MH "intracranial embolism and thrombosis" OR TI "ischemic stroke" OR AB "ischemic stroke" OR TI "hemorrhagic stroke" OR AB "hemorrhagic stroke" OR TI "brain ischemia" OR AB "brain ischemia" OR TI "cerebral ischemia" OR AB "cerebral ischemia" OR TI "brain infarct*" OR AB "brain infarct*" OR TI "brain vascular accident*" OR AB "brain vascular accident*" OR TI "cerebral stroke*" OR AB "cerebral stroke*" OR TI "cerebral infarct*" OR AB "cerebral infarct*" OR TI "cerebral accident*" OR AB "cerebral accident*" OR TI "ischemic stroke*" OR AB "ischemic stroke*" OR TI "intracranial infarct*" OR AB "intracranial infarct*" OR TI "lacunar stroke*" OR AB "lacunar stroke*" OR TI "lacunar infarct*" OR AB "lacunar infarct*" OR TI "stroke*" OR AB "stroke*" OR TI "small vessel stroke*" OR AB "small vessel stroke*" OR TI "cerebral artery disease*" OR AB "cerebral artery disease*" OR TI "intracranial artery disease*" OR AB "intracranial artery disease*" OR TI "brain embol*" OR AB "brain embol*" OR TI "cerebral embol*" OR AB "cerebral embol*" OR TI "cerebral thromb*" OR AB "cerebral thromb*" OR TI "intracranial embol*" OR AB "intracranial embol*" OR TI "intracranial thromb*" OR AB "intracranial thromb*" OR TI "cardioembolic stroke*" OR AB "cardioembolic stroke*" OR TI "embolic stroke*" OR AB "embolic stroke*" OR TI "thrombotic stroke*" OR AB "thrombotic stroke*" ) | 448,811 |
|  | #2 | ( MH "multiomics" OR MH "genomics" OR MH "transcriptome" OR MH "proteomics" OR MH "proteome" OR MH "metabolomics" OR MH "metabolome" OR TI "multi-omics" OR AB "multi-omics" OR TI "multiomics" OR AB "multiomics" OR TI "integrative omics" OR AB "integrative omics" OR TI "transcriptomics" OR AB "transcriptomics" OR TI "transcriptome profil*" OR AB "transcriptome profil*" OR TI "proteomics" OR AB "proteomics" OR TI "metabolomics" OR AB "metabolomics" OR TI "bioinformatic analys*" OR AB "bioinformatic analys*" OR TI "whole genome" OR AB "whole genome" OR TI "whole exome" OR AB "whole exome" OR TI "genome-wide" OR AB "genome-wide" OR TI "RNAseq" OR AB "RNAseq" OR TI "RNA-seq" OR AB "RNA-seq" OR TI "microarray" OR AB "microarray" OR TI "transcriptom*" OR AB "transcriptom*" OR TI "genomic*" OR AB "genomic*" OR TI "epigenomic*" OR AB "epigenomic*" OR TI "proteomic*" OR AB "proteomic*" OR TI "metabolomic*" OR AB "metabolomic*" OR TI "omic*" OR AB "omic*" OR TI "biomarker*" OR AB "biomarker*" OR TI "bioinformatics" OR AB "bioinformatics" ) | 1,573,311 |
|  | #3 | ( MH "machine learning" OR MH "artificial intelligence" OR TI "machine learning" OR AB "machine learning" OR TI "artificial intelligence" OR AB "artificial intelligence" OR TI "deep learning" OR AB "deep learning" OR TI "transfer learning" OR AB "transfer learning" OR TI "support vector machine" OR AB "support vector machine" OR TI "XGBoost" OR AB "XGBoost" OR TI "random forest" OR AB "random forest" OR TI "gradient boosting machine" OR AB "gradient boosting machine" OR TI "ensemble learning" OR AB "ensemble learning" OR TI "neural network" OR AB "neural network" OR TI "neural networks" OR AB "neural networks" OR TI "multilayer perceptron" OR AB "multilayer perceptron" OR TI "k nearest neighbor" OR AB "k nearest neighbor" OR TI "Adaboost" OR AB "Adaboost" OR TI "ResNet" OR AB "ResNet" OR TI "decision tree" OR AB "decision tree" OR TI "bayesian network" OR AB "bayesian network" OR TI "naive bayesian" OR AB "naive bayesian" OR TI "risk model" OR AB "risk model" OR TI "prediction model" OR AB "prediction model" OR TI "nomogram" OR AB "nomogram" ) | 456,345 |
|  |  | Filters: Full text, from 2000/1/1 – to 2025/07/31 | **496** |
| EMBASE | #1 | ('stroke'/exp OR 'brain ischemia'/exp OR 'brain ischemia':ti,ab OR 'cerebral ischemia':ti,ab OR 'ischemic stroke':ti,ab OR 'hemorrhagic stroke':ti,ab OR 'brain infarct*':ti,ab OR 'brain vascular accident*':ti,ab OR 'cerebral stroke*':ti,ab OR 'cerebral infarct*':ti,ab OR 'cerebral accident*':ti,ab OR 'intracranial infarct*':ti,ab OR 'lacunar stroke*':ti,ab OR 'lacunar infarct*':ti,ab OR 'small vessel stroke*':ti,ab OR 'intracranial arteriosclerosis'/exp OR 'cerebral arterial disease' OR 'cerebral artery disease*':ti,ab OR 'intracranial artery disease*':ti,ab OR 'intracranial embolism'/exp OR 'brain embol*':ti,ab OR 'cerebral embol*':ti,ab OR 'cerebral thromb*':ti,ab OR 'intracranial embol*':ti,ab OR 'intracranial thromb*':ti,ab OR 'cardioembolic stroke*':ti,ab OR 'embolic stroke*':ti,ab OR 'thrombotic stroke*':ti,ab) AND [humans]/lim AND [01-01-2000]/sd NOT [01-08-2025]/sd | 578,512 |
|  | #2 | ('multi omics'/exp OR 'multi-omics':ti,ab OR 'multiomics':ti,ab OR 'integrative omics'/exp OR 'integrative omics':ti,ab OR 'genomics'/exp OR 'transcriptome'/exp OR 'transcriptomics':ti,ab OR 'transcriptome profil*':ti,ab OR 'proteomics'/exp OR 'proteomics':ti,ab OR 'proteome'/exp OR 'metabolomics'/exp OR 'metabolome'/exp OR 'metabolomics':ti,ab OR 'bioinformatic analys*':ti,ab OR 'whole genome':ti,ab OR 'whole exome':ti,ab OR 'genome-wide':ti,ab OR 'rnaseq':ti,ab OR 'rna-seq':ti,ab OR 'microarray':ti,ab OR 'transcriptom*':ti,ab OR 'genomic*':ti,ab OR 'epigenomic*':ti,ab OR 'proteomic*':ti,ab OR 'metabolomic*':ti,ab OR 'omic*':ti,ab) AND [humans]/lim AND [01-01-2000]/sd NOT [01-08-2025]/sd | 872,588 |
|  | #3 | ('machine learning'/exp OR 'machine learning':ti,ab OR 'artificial intelligence':ti,ab OR 'deep learning':ti,ab OR 'transfer learning':ti,ab OR 'support vector machine':ti,ab OR 'xgboost':ti,ab OR 'random forest':ti,ab OR 'gradient boosting machine':ti,ab OR 'ensemble learning':ti,ab OR 'neural network':ti,ab OR 'multilayer perceptron':ti,ab OR 'k nearest neighbor':ti,ab OR 'adaboost':ti,ab OR 'resnet':ti,ab OR 'decision tree':ti,ab OR 'bayesian network':ti,ab OR 'naive bayesian':ti,ab OR 'risk model':ti,ab OR 'prediction model':ti,ab OR 'nomogram':ti,ab) AND [humans]/lim AND [01-01-2000]/sd NOT [01-08-2025]/sd | 540,046 |
|  |  | AND [humans]/lim AND [01-01-2000]/sd NOT [01-08-2025]/sd |  |
|  |  | #1 AND #2 AND #3 | **504** |
| CINAHL | #1 | ( MH "brain ischemia" OR MH "stroke" OR MH "intracranial arteriosclerosis" OR MH "cerebral arterial diseases" OR MH "intracranial embolism and thrombosis" OR TI "ischemic stroke" OR AB "ischemic stroke" OR TI "hemorrhagic stroke" OR AB "hemorrhagic stroke" OR TI "brain ischemia" OR AB "brain ischemia" OR TI "cerebral ischemia" OR AB "cerebral ischemia" OR TI "brain infarct*" OR AB "brain infarct*" OR TI "brain vascular accident*" OR AB "brain vascular accident*" OR TI "cerebral stroke*" OR AB "cerebral stroke*" OR TI "cerebral infarct*" OR AB "cerebral infarct*" OR TI "cerebral accident*" OR AB "cerebral accident*" OR TI "ischemic stroke*" OR AB "ischemic stroke*" OR TI "intracranial infarct*" OR AB "intracranial infarct*" OR TI "lacunar stroke*" OR AB "lacunar stroke*" OR TI "lacunar infarct*" OR AB "lacunar infarct*" OR TI "stroke*" OR AB "stroke*" OR TI "small vessel stroke*" OR AB "small vessel stroke*" OR TI "cerebral artery disease*" OR AB "cerebral artery disease*" OR TI "intracranial artery disease*" OR AB "intracranial artery disease*" OR TI "brain embol*" OR AB "brain embol*" OR TI "cerebral embol*" OR AB "cerebral embol*" OR TI "cerebral thromb*" OR AB "cerebral thromb*" OR TI "intracranial embol*" OR AB "intracranial embol*" OR TI "intracranial thromb*" OR AB "intracranial thromb*" OR TI "cardioembolic stroke*" OR AB "cardioembolic stroke*" OR TI "embolic stroke*" OR AB "embolic stroke*" OR TI "thrombotic stroke*" OR AB "thrombotic stroke*" ) | 146,697 |
|  | #2 | ( MH "multiomics" OR MH "genomics" OR MH "transcriptome" OR MH "proteomics" OR MH "proteome" OR MH "metabolomics" OR MH "metabolome" OR TI "multi-omics" OR AB "multi-omics" OR TI "multiomics" OR AB "multiomics" OR TI "integrative omics" OR AB "integrative omics" OR TI "transcriptomics" OR AB "transcriptomics" OR TI "transcriptome profil*" OR AB "transcriptome profil*" OR TI "proteomics" OR AB "proteomics" OR TI "metabolomics" OR AB "metabolomics" OR TI "bioinformatic analys*" OR AB "bioinformatic analys*" OR TI "whole genome" OR AB "whole genome" OR TI "whole exome" OR AB "whole exome" OR TI "genome-wide" OR AB "genome-wide" OR TI "RNAseq" OR AB "RNAseq" OR TI "RNA-seq" OR AB "RNA-seq" OR TI "microarray" OR AB "microarray" OR TI "transcriptom*" OR AB "transcriptom*" OR TI "genomic*" OR AB "genomic*" OR TI "epigenomic*" OR AB "epigenomic*" OR TI "proteomic*" OR AB "proteomic*" OR TI "metabolomic*" OR AB "metabolomic*" OR TI "omic*" OR AB "omic*" OR TI "biomarker*" OR AB "biomarker*" OR TI "bioinformatics" OR AB "bioinformatics" ) | 150,275 |
|  | #3 | ( MH "machine learning" OR MH "artificial intelligence" OR TI "machine learning" OR AB "machine learning" OR TI "artificial intelligence" OR AB "artificial intelligence" OR TI "deep learning" OR AB "deep learning" OR TI "transfer learning" OR AB "transfer learning" OR TI "support vector machine" OR AB "support vector machine" OR TI "XGBoost" OR AB "XGBoost" OR TI "random forest" OR AB "random forest" OR TI "gradient boosting machine" OR AB "gradient boosting machine" OR TI "ensemble learning" OR AB "ensemble learning" OR TI "neural network" OR AB "neural network" OR TI "neural networks" OR AB "neural networks" OR TI "multilayer perceptron" OR AB "multilayer perceptron" OR TI "k nearest neighbor" OR AB "k nearest neighbor" OR TI "Adaboost" OR AB "Adaboost" OR TI "ResNet" OR AB "ResNet" OR TI "decision tree" OR AB "decision tree" OR TI "bayesian network" OR AB "bayesian network" OR TI "naive bayesian" OR AB "naive bayesian" OR TI "risk model" OR AB "risk model" OR TI "prediction model" OR AB "prediction model" OR TI "nomogram" OR AB "nomogram" ) | 71,391 |
|  |  | Filters: Full text |  |
|  |  | #1 AND #2 AND #3 | **38** |
| Web of Science | #1 | (TS=("ischemic stroke" OR "hemorrhagic stroke" OR "brain ischemia" OR "cerebral ischemia" OR "brain infarct*" OR "brain vascular accident*" OR "cerebral stroke*" OR "cerebral infarct*" OR "cerebral accident*" OR "ischemic stroke*" OR "intracranial infarct*" OR "lacunar stroke*" OR "lacunar infarct*" OR "stroke*" OR "small vessel stroke*" OR "cerebral artery disease*" OR "intracranial artery disease*" OR "brain embol*" OR "cerebral embol*" OR "cerebral thromb*" OR "intracranial embol*" OR "intracranial thromb*" OR "cardioembolic stroke*" OR "embolic stroke*" OR "thrombotic stroke*")) | 566,556 |
|  | #2 | (TS=("multi-omics" OR "multiomics" OR "integrative omics" OR "transcriptomics" OR "transcriptome profil*" OR "proteomics" OR "metabolomics" OR "bioinformatic analys*" OR "whole genome" OR "whole exome" OR "genome-wide" OR "RNAseq" OR "RNA-seq" OR "microarray" OR "transcriptom*" OR "genomic*" OR "epigenomic*" OR "proteomic*" OR "metabolomic*" OR "omic*" OR "biomarker*" OR "bioinformatics")) | 1,902,557 |
|  | #3 | (TS=( "machine learning" OR "artificial intelligence" OR "deep learning" OR "transfer learning" OR "support vector machine" OR "XGBoost" OR "random forest" OR "gradient boosting machine" OR "ensemble learning" OR "neural network" OR "multilayer perceptron" OR "k nearest neighbor" OR "Adaboost" OR "ResNet" OR "decision tree" OR "bayesian network" OR "naive bayesian" OR "risk model" OR "prediction model" OR "nomogram")) | 1,645,935 |
|  |  | Filters: Article, from 2000/1/1 – to 2025/07/31 |  |
|  |  | #1 AND #2 AND #3 | **775** |
| SCOPUS | #1 | (TITLE-ABS-KEY ( "stroke" OR "brain ischemia" OR "cerebral ischemia" OR "ischemic stroke" OR "hemorrhagic stroke" OR "brain infarct*" OR "cerebral infarct*" OR "lacunar stroke*" OR "lacunar infarct*" OR "small vessel stroke*" OR "cerebral artery disease*" OR "intracranial artery disease*" OR "cerebral embol*" OR "brain embol*" OR "intracranial embol*" OR "cardioembolic stroke*" OR "thrombotic stroke*" )) | 725,207 |
|  | #2 | (TITLE-ABS-KEY ( "multi-omics" OR "multiomics" OR "integrative omics" OR "genomics" OR "transcriptomics" OR "transcriptome profiling" OR "proteomics" OR "metabolomics" OR "epigenomics" OR "whole genome" OR "whole exome" OR "genome-wide" OR "RNAseq" OR "RNA-seq" OR "microarray" )) | 1,054,404 |
|  | #3 | (TITLE-ABS-KEY ( "machine learning" OR "artificial intelligence" OR "deep learning" OR "transfer learning" OR "support vector machine" OR "random forest" OR "XGBoost" OR "gradient boosting" OR "ensemble learning" OR "neural network" OR "decision tree" OR "prediction model" OR "nomogram" )) | 2,955,088 |
|  |  | Filters: Article, from 2000-2025 |  |
|  |  | #1 AND #2 AND #3 | **280** |
| Cochrane CENTRAL | #1 | ((ischemic stroke OR hemorrhagic stroke OR brain ischemia OR cerebral ischemia OR stroke OR brain infarct* OR brain vascular accident* OR cerebral stroke* OR cerebral infarct* OR cerebral accident* OR ischemic stroke* OR intracranial infarct* OR lacunar stroke* OR lacunar infarct* OR small vessel stroke* OR intracranial arteriosclerosis OR cerebral arterial disease* OR cerebral artery disease* OR intracranial artery disease* OR intracranial embol* OR brain embol* OR cerebral embol* OR cerebral thromb* OR intracranial thromb* OR cardioembolic stroke* OR embolic stroke* OR thrombotic stroke*) in Title Abstract Keyword) | 87,284 |
|  | #2 | ((multi-omics OR multiomics OR integrative omics OR genomics OR transcriptomics OR transcriptome profil* OR proteomics OR metabolomics OR metabolome OR bioinformatic analys* OR whole genome OR whole exome OR genome-wide OR RNAseq OR RNA-seq OR microarray OR transcriptom* OR genomic* OR epigenomic* OR proteomic* OR metabolomic* OR omic* OR biomarker* OR bioinformatics) in Title Abstract Keyword) | 75,612 |
|  | #3 | ((machine learning OR artificial intelligence OR deep learning OR transfer learning OR support vector machine OR XGBoost OR random forest OR gradient boosting machine OR ensemble learning OR neural network OR multilayer perceptron OR k nearest neighbor OR Adaboost OR ResNet OR decision tree OR bayesian network OR naive bayesian OR risk model OR prediction model OR nomogram) in Title Abstract Keyword) | 113,665 |
|  |  | Filters: from Jan 2000-Jul 2025, in Trials |  |
|  |  | #1 AND #2 AND #3 | **301** |
| ACM | #1 | [All: stroke] OR [All: "brain ischemia"] OR [All: "cerebral ischemia"] OR [All: "ischemic stroke"] OR [All: "hemorrhagic stroke"] OR [All: "brain infarct*"] OR [All: "brain vascular accident*"] OR [All: "cerebral stroke*"] OR [All: "cerebral infarct*"] OR [All: "cerebral accident*"] OR [All: "intracranial infarct*"] OR [All: "lacunar stroke*"] OR [All: "lacunar infarct*"] OR [All: "small vessel stroke*"] OR [All: "cerebral artery disease*"] OR [All: "intracranial artery disease*"] OR [All: "brain embol*"] OR [All: "cerebral embol*"] OR [All: "cerebral thromb*"] OR [All: "intracranial embol*"] OR [All: "intracranial thromb*"] OR [All: "cardioembolic stroke*"] OR [All: "embolic stroke*"] OR [All: "thrombotic stroke*"] applied filters Journals, Research Article, from 2000-2025 | 1,700 |
|  | #2 | [All: "multi-omics"] OR [All: "multiomics"] OR [All: "integrative omics"] OR [All: "genomics"] OR [All: "transcriptomics"] OR [All: "transcriptome profil*"] OR [All: "proteomics"] OR [All: "metabolomics"] OR [All: "metabolome"] OR [All: "bioinformatic analys*"] OR [All: "whole genome"] OR [All: "whole exome"] OR [All: "genome-wide"] OR [All: "rnaseq"] OR [All: "rna-seq"] OR [All: "microarray"] OR [All: "transcriptom*"] OR [All: "genomic*"] OR [All: "epigenomic*"] OR [All: "proteomic*"] OR [All: "metabolomic*"] OR [All: "omic*"]  applied filters Journals, Research Article, from 2000-2025 | 1,445 |
|  | #3 | [All: "machine learning"] OR [All: "artificial intelligence"] OR [All: "deep learning"] OR [All: "transfer learning"] OR [All: "support vector machine"] OR [All: "xgboost"] OR [All: "random forest"] OR [All: "gradient boosting machine"] OR [All: "ensemble learning"] OR [All: "neural network"] OR [All: "multilayer perceptron"] OR [All: "k nearest neighbor"] OR [All: "adaboost"] OR [All: "resnet"] OR [All: "decision tree"] OR [All: "bayesian network"] OR [All: "naive bayesian"] OR [All: "risk model"] OR [All: "prediction model"] OR [All: "nomogram"]  applied filters Journals, Research Article, from 2000-2025 | 29,841 |
|  |  | Filters: Journals, Research Article, from January 2000-July 2025 |  |
|  |  | #1 AND #2 AND #3 | **17** |
| IEEE Xplore |  | ("All Metadata":stroke* OR "All Metadata":infarct*) AND ("All Metadata":omics* OR "All Metadata":biomarker*) AND ("All Metadata":machine learning OR "All Metadata":deep learning OR "All Metadata":artificial intelligence OR "All Metadata":ensemble learning OR "All Metadata":prediction*) |  |
|  |  | Filters Applied: Journals, Early Access Articles, 2000 - 2025 | **36** |
