# Supplementary material for: Machine Learning for Predicting Stroke Risk Stratification Using Multiomics Data: Systematic Review
Source: J Med Internet Res. 2026 Feb 19;28:e85654. doi: 10.2196/85654 (PMC12963974; doi:10.2196/85654)
Supplement: Multimedia Appendix 3 [file jmir_v28i1e85654_app3.docx]

Multimedia Appendix. Critical appraisal

| 1. PROBAST: Risk of bias and applicability assessment tool. | | | | | | | | | |
| --- | --- | --- | --- | --- | --- | --- | --- | --- | --- |
| Study No. | Risk of bias | | | | Applicability | | | Overall | |
|  | Participants | Predictors | Outcome | Analysis | Participants | Predictors | Outcome | Risk of bias | Applicability |
| [35] | + | + | + | - | + | + | + | ? | + |
| [36] | + | + | + | ? | + | + | + | + | + |
| [37] | + | + | + | + | + | + | + | + | + |
| [38] | + | + | + | - | + | + | + | - | + |
| [39] | + | + | + | - | + | + | + | - | + |
| [40] | + | + | + | + | + | + | + | + | + |
| [41] | + | + | + | + | + | + | + | + | + |

Abbreviations: + =indicates low bias/low concern regarding applicability; - = indicates high bias/ high concern regarding applicability; ? = indicates unclear bias/ unclear concern regarding applicabilit

2. MINimum Information for Medical AI Reporting (MINIMAR) checklist .

| MINIMAR reporting element | [35] | [36] | [37] | [38] | [39] | [40] | [41] |
| --- | --- | --- | --- | --- | --- | --- | --- |
| 1. Study population and setting |  |  |  |  |  |  |  |
| Population | Y | Y | Y | Y | Y | Y | Y |
| Study setting | Y | Y | Y | Y | Y | Y | Y |
| Data source | Y | Y | Y | Y | Y | Y | Y |
| Cohort selection | Y | Y | Y | Y | Y | Y | Y |
| 2. Patient demographic characteristics |  |  |  |  |  |  |  |
| Age | N | Y | Y | Y | Y | Y | Y |
| Sex | N | Y | Y | Y | Y | Y | Y |
| Race | P | N | N | N | N | N | Y |
| Ethnicity | N | N | N | N | N | P | P |
| Socioeconomic status | N | N | N | N | N | N | P |
| 3. Model architecture |  |  |  |  |  |  |  |
| Model output | Y | Y | Y | Y | Y | Y | Y |
| Target user | P | P | P | P | P | P | P |
| Data splitting | Y | Y | Y | Y | Y | Y | Y |
| Gold standard | P | Y | Y | Y | Y | Y | Y |
| Model task | Y | Y | Y | Y | Y | Y | Y |
| Model architecture | Y | Y | Y | Y | Y | Y | Y |
| Features | Y | Y | Y | Y | Y | Y | Y |
| Missingness | N | P | P | N | P | Y | Y |
| 4. Model evaluation |  |  |  |  |  |  |  |
| Optimization | P | P | Y | P | Y | Y | Y |
| Internal model validation | Y | Y | Y | Y | Y | Y | Y |
| External model validation | Y | Y | N | N | N | Y | Y |
| Transparency | P | P | P | P | Y | P | P |
| Score | 11/21 | 13/21 | 14/21 | 13/21 | 15/21 | 16/21 | 17/21 |

Abbreviations; Y= Yes for reported; P= Poorly reported; N= Not reported; x= not applicable
